# Defect-Mediated Atomic Layer Etching Processes on Cl–Si(100): An Atomistic Insight

**DOI:** 10.1021/acs.jpcc.3c05378

**Published:** 2023-10-23

**Authors:** Peizhi Wang, Fengzhou Fang

**Affiliations:** † Centre of Micro/Nano Manufacturing Technology (MNMT-Dublin), University College Dublin, Dublin D4, Ireland; ‡ State Key Laboratory of Precision Measuring Technology and Instruments, Laboratory of Micro/Nano Manufacturing Technology (MNMT), Tianjin University, Tianjin 300072, China

## Abstract

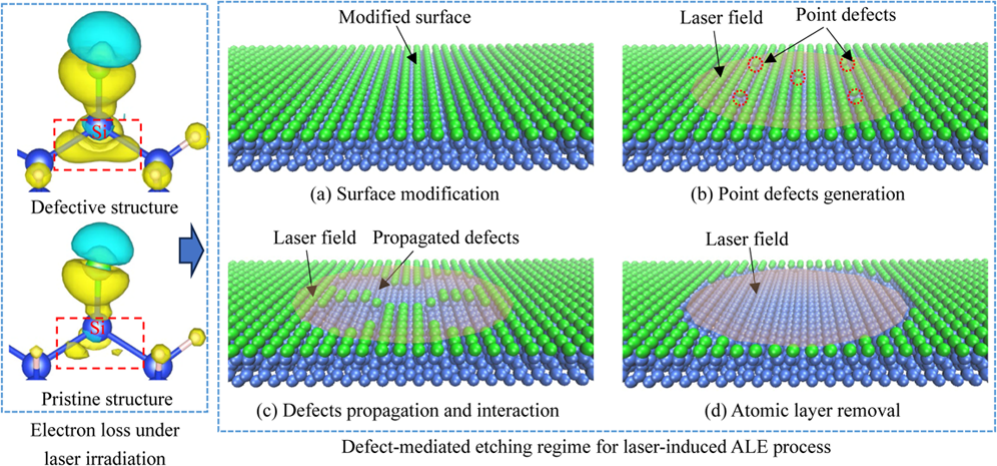

Defects play a significant role in atomic layer etching (ALE) processes; however, a fundamental understanding at the atomic level is still lacking. To bridge this knowledge gap, this study investigated the role of point defects in the laser-induced ALE of Cl–Si(100) using density functional theory (DFT) and real-time time-dependent DFT calculations. In the calculations, both the pristine surface and the defective surface were considered for comparative analysis. The key finding is the enhanced desorption of SiCl molecules, facilitated by point defects under laser pulse irradiation. The presence of point defects was found to effectively reduce both the desorption energy barrier and the laser intensity threshold required for desorption. Additionally, extra defective levels within the band gap were observed through the density-of-state diagram. Based on these findings, a defect-mediated etching regime was proposed to elucidate the layer-by-layer etching process. This study provides atomistic insight into understanding the role of defects in laser-induced ALE processes. The presence of point defects can enhance the etching selectivity between the topmost layer and the underlying layers, thereby contributing to highly efficient and damage-free etching processes through the defect-mediated etching mechanism.

## 1 Introduction

Atomic layer etching (ALE), as the most advanced etching technique, has garnered significant interest owing to its potential applications in the continual miniaturization of semiconductor devices in atomic and close-to-atomic scale manufacturing (ACSM) era.^1−10^ In the framework of ALE, the atomically precise etching process is ensured through sequential and self-limiting reactions. Taking the most extensively studied case of silicon (Si) as an example, the topmost Si layer of the substrate is first modified by chlorine (Cl) during the initial step of ALE. Subsequently, the modified layer undergoes selective removal using various energy fields, such as plasma (plasma-based ALE), high temperature (thermal ALE), and laser (laser-induced ALE). Despite the great success achieved by the plasma and thermal ALE approaches, several challenges persist, such as the limited efficiency of high-temperature processes and the occurrence of surface damage caused by high-energy ions and unwanted “background” etching.^11,12^ As a complementary approach, laser-induced atomic layer etching (ALE) was introduced in the early 1990s. The initial investigations involved the reaction of Cl-containing gases with Si surfaces under laser irradiation, demonstrating that the etching rate could be reduced to nearly a monatomic layer per pulse.^13,14^ Subsequently, the laser-induced ALE technique was employed for the precision etching of GaAs using Cl_2_ as the modifying precursor.^15,16^ Recently, a two-step pulsed laser chemical-assisted ALE process was proposed to enhance the controllability and practicality of Si etching.^17^ In this approach, a nanosecond laser was utilized for surface modification, while a picosecond pulsed laser was employed for material removal. Beyond bulk material etching, the laser-induced ALE technique has found applications in layer thinning of layered materials such as MoS_2_, MoTe_2_, and others.^18−21^ The use of lasers in the ALE process has relatively lower vacuum requirements when compared with plasma-based methods. Furthermore, it offers spatial selectivity, minimizing damage to adjacent structures, especially when the optical system is well-designed and developed.

As an essential component of research on ALE, the etching mechanisms on various materials have undergone extensive investigations, employing both experimental and simulation methods. Density functional theory (DFT) has been employed to scrutinize the microscopic interactions of precursors with the topmost substrate layer, revealing that the bonds between the top layer and the underlying layers can be selectively weakened using appropriate precursors.^22−24^ For example, the desorption energy of a silicon atom can be reduced from 7.40 to 8.46 eV to 1.4–3.2 eV (if SiCl_2_ is formed) and 4.52–5.74 eV (if SiCl is formed). To explore ion collisions with the modified layer, molecular dynamics (MD) simulations have been used to simulate plasma-based ALE of Si and Ge.^25−27^ These simulations have revealed that achieving ideal etching processes is challenging, resulting in the formation of amorphous and damaged layers due to high-energy ion bombardment. Experimental observations have identified an ion energy window beyond which the self-limiting process is disrupted.^3,28^ This window has a width of 20 eV in Si ALE and 40 eV in amorphous carbon ALE. Besides, the etching mechanisms of thermal ALE have been categorized into fluorination, ligand exchange, conversion, oxidation, and halogenation mechanisms, among others, as summarized in reviews.^29,30^ Concerning laser-induced ALE, real-time time-dependent DFT calculations have been employed to investigate the interaction between chlorinated Si layers and pulsed lasers. It was found that electron loss between Si–Si bonds leads to the desorption of SiCl from the surface.^31,32^

However, the studies above primarily focused on the removal process from pristine surfaces while neglecting defects generated during synthesis and manufacturing processes.^33−35^ Particularly in the context of the laser-induced ALE process, scanning tunneling microscopy (STM) images have revealed that defects are initially generated and subsequently propagate horizontally, resulting in the atomic layer removal of GaAs under laser pulses.^36^ Such defects can significantly impact the electrical, magnetic, optical, and catalytic properties of materials, thereby influencing material removal and surface integrity.^37,38^ Nevertheless, there remains a knowledge gap in the fundamental understanding of the role of defects in ALE processes, especially in the case of Si, the most common semiconductor material.

To address the above-mentioned challenges, this study investigated the role of point defects in laser-induced ALE of Cl–Si (100) at an atomic level. DFT calculations were first employed to determine the relaxed geometry of defective structures as well as to calculate the desorption energy barrier and electronic structures. Furthermore, real-time time-dependent DFT (rt-TDDFT) calculations were utilized to investigate the desorption dynamics of both the pristine and defective structures under laser pulses. The findings revealed that the presence of defects leads to a reduction in the desorption energy barrier and lowers the laser intensity threshold required for the desorption of SiCl molecules. Based on these findings, a defect-mediated etching regime was proposed to explain the layer-by-layer etching process.

## 2 Methodology

The geometry relaxation, desorption energy barrier, and electronic structures of defective and pristine Si (100) were computed by DFT calculations using Quantum Espresso software. The Perdew–Burke–Ernzerhof (PBE) exchange–correlation functional was used for calculation.^39,40^ A kinetic energy cutoff of 50 Ry (200 Ry for charge density) was selected for the wave function. The total energy convergence threshold and force convergence threshold for structure optimization were 5 × 10^–5^ Ry and 5 × 10^–4^ Ry/Bohr, respectively. The convergence threshold for self-consistent field calculations was 1 × 10^–7^ Ry. The calculations utilized a (4 × 2)-Si (100) unit cell comprising nine Si layers. A Monkhorst–Pack 2 × 4 × 1 *k*-point mesh was employed in reciprocal space.^41^ The topmost Si layer was modified with Cl atoms, and a vacuum region of 20 Å was incorporated along the vertical direction to prevent interactions between the top and bottom layers under periodic boundary conditions. The remaining dangling bonds of Si were passivated by hydrogen (H) atoms. In the calculations, a total of nine Si layers were used, with the bottom five Si layers and the H layer being fixed.

The desorption dynamics under laser pulses were investigated by solving time-dependent Kohn–Sham equations using the rt-TDDFT code implemented within Octopus.^42^ The Hartwigsen–Goedecker–Hutter (HGH) pseudopotentials with the local density approximation functional (LDA) method were employed.^43^ The selected pseudopotentials and cluster size have been tested in our previous research.^32^ The simulation box had dimensions of 20 × 20 × 20 Å^3^ with a mesh size of 0.2 Å. The time evolution was computed over 150,000 steps with a time step of 0.002 ℏ/eV (approximately 0.00132 fs) using the enforced time-reversal symmetry (ETRS) propagating algorithm.^44^ The external laser pulses were polarized along the vertical direction and characterized by a Gaussian-shape electric field *E*_laser_(*t*) = *E*_0_ sin(ω*t*) exp(−(*t* – *t*_0_)^2^/(2τ_0_^2^)), where the pulse width τ_0_ was set to 40 ℏ/eV (26.32 fs) and the maximum laser intensity was reached at *t*_0_= 100 ℏ/eV (65.80 fs); the magnitude of field *E*_0_ and frequency/wavelength of photons ω are given in Section 3.2.

## 3 Results and Discussion

### 3.1 Desorption Energy Barrier and Electronic Structures

**Figure 1:**
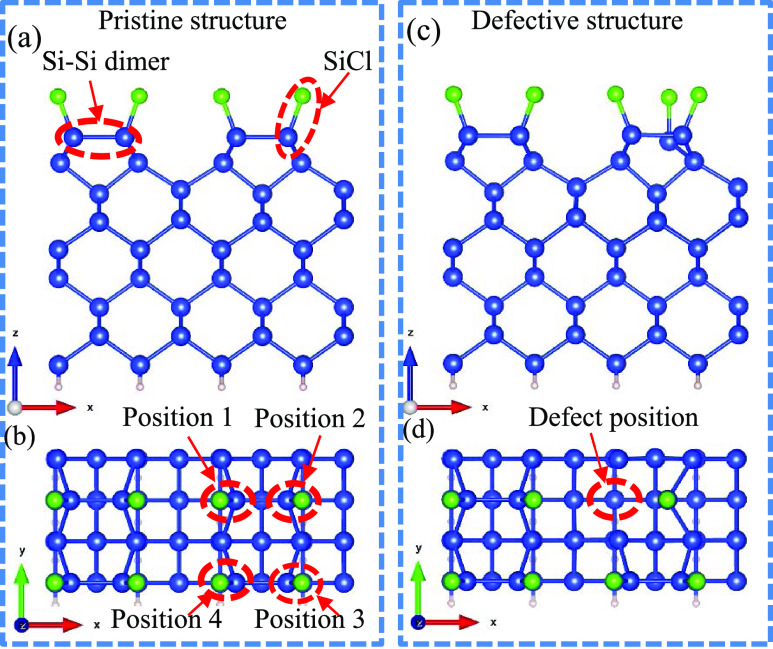
Front (a, c) and top (b, d) views of the geometry structures of defect-free pristine and defective Cl–Si (100) slabs for DFT calculations. Blue, green, and red atoms are Si, Cl, and H, respectively.

Figure 1 shows the relaxed geometry structures of defect-free (pristine) and defective Cl:Si (100) slabs. Upon relaxation, the topmost Si layer undergoes a 2 × 1-type surface reconstruction, resulting in the formation of Si–Si dimers. The adsorbed Cl atom forms a bond with the Si dimer atom, exhibiting a bond length of 2.0 Å and a tilt angle of 20° off the surface normal. These values are consistent with those reported in the literature.^45^ It is assumed that the Si atom at position 1 is removed during the ALE process, resulting in the occurrence of a point defect at this position, as shown in Figure 1c,d. This Si vacancy breaks the Si–Si dimer between positions 1 and 2, causing the SiCl molecule at position 2 to move toward the defect position due to the repulsion between the two dimer Si atoms being released.

**Figure 2:**
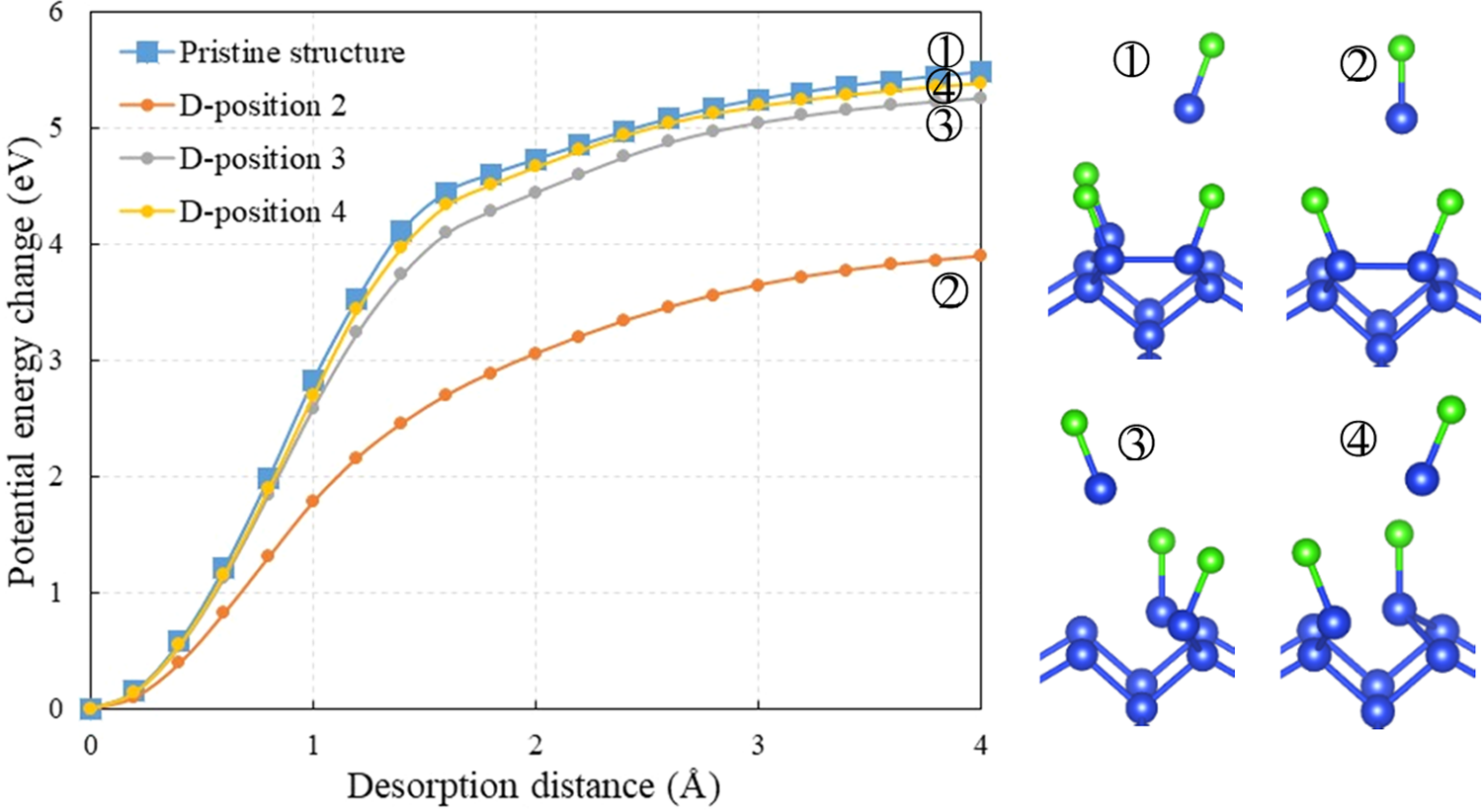
Ground-state potential energy curves during the desorption of the SiCl molecule from pristine and defective structures. “D” represents the defective structure. The panels ①–④ are the local structures of desorbed SiCl with a desorption distance of 4 Å.

The effects of the point defect on the ground-state potential energy curves during the desorption of SiCl are depicted in Figure 2. These potential energy curves were generated by calculating the system’s energy as the SiCl molecule moved away from the surface along the *z*-axis (perpendicular to the surface). The horizontal axis represents the displacement of the SiCl molecule from its initial position. It is evident that the pristine structure exhibits the highest desorption energy barrier, which measures 5.48 eV, and the desorption energy barrier is defined as the energy required to move the adsorbed atoms or molecules away from the surface. However, the presence of the point defect significantly reduces this barrier to 3.21 eV for the SiCl molecule located at position 2. This reduction is primarily attributed to the breakage of the Si–Si dimer bond between positions 1 and 2 caused by the defect. In contrast, the desorption energy barriers at the nearby dimer sites at positions 3 and 4 were calculated to be 5.41 and 4.47 eV, respectively, and they are less affected by the point defect. This observation aligns with the fact that the geometry structure at these two positions experiences lesser perturbations.

**Figure 3:**
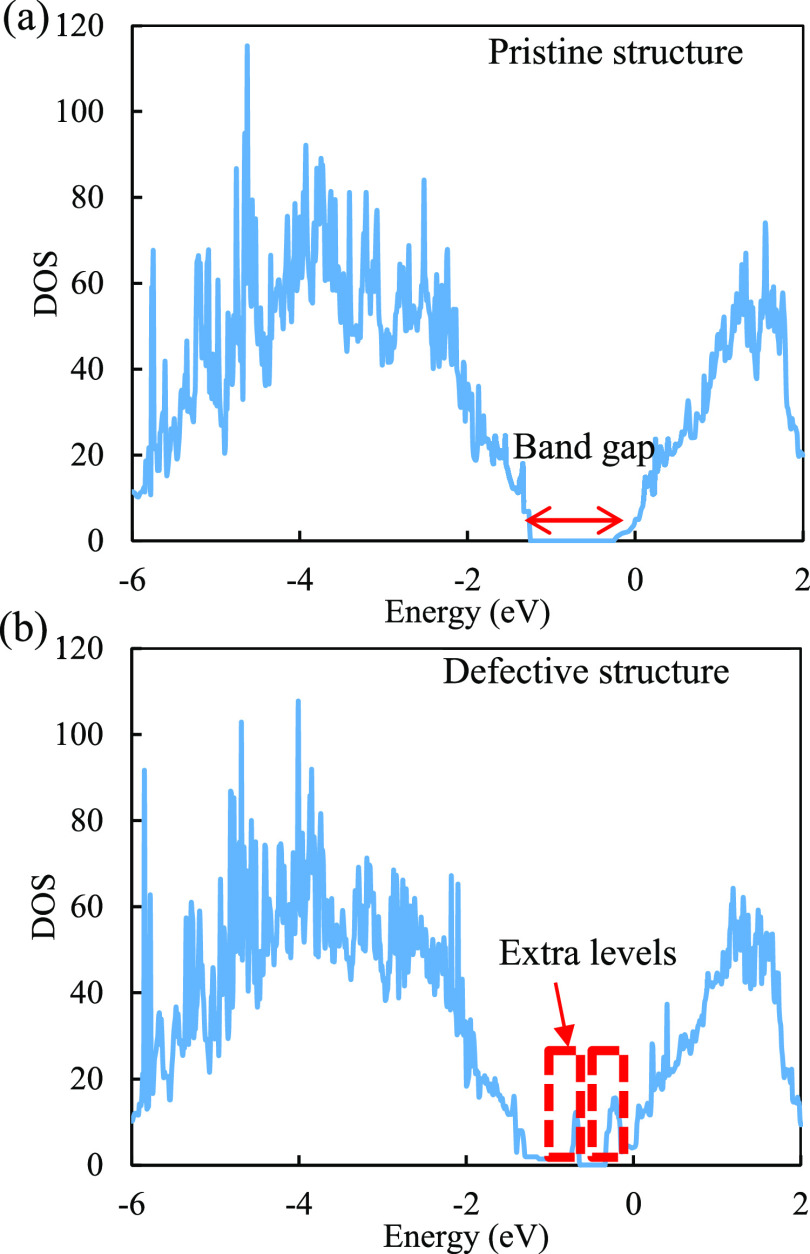
Density of states (DOS) of the (a) pristine Cl–Si (100) structure and (b) defective Cl–Si (100) structure.

Additionally, the density of states (DOS) was calculated to investigate the effect of point defects on electronic structures of Cl–Si (100), as shown in Figure 3. In order to achieve more accurate results, a Monkhorst–Pack 6 × 12 × 1 *k*-point mesh was applied. DOS far from the band gap were excluded from the analysis.

The pristine structure exhibits a band gap of 0.90 eV, as shown in Figure 3a, which is consistent with the reported value in the literature.^46^ When compared to the pristine structure, the DOS curve of the defective structure displays similar peak values and positions within the valence band (VB) and conduction band (CB), except for the region at the edge of VB and CB. The primary distinction is the presence of additional levels in the band gap due to the point defect. This is because the periodic potential field near this point is disturbed due to the presence of a point defect. These defect-induced extra levels have also been observed in other materials, such as Al_2_O_3_, Bi_2_Te_3_, and SnC nanosheets.^47−49^ The presence of these additional energy levels can serve as a transition state, facilitating potential reactions and enhancing the desorption process.

### 3.2 Desorption Dynamics

**Figure 4:**
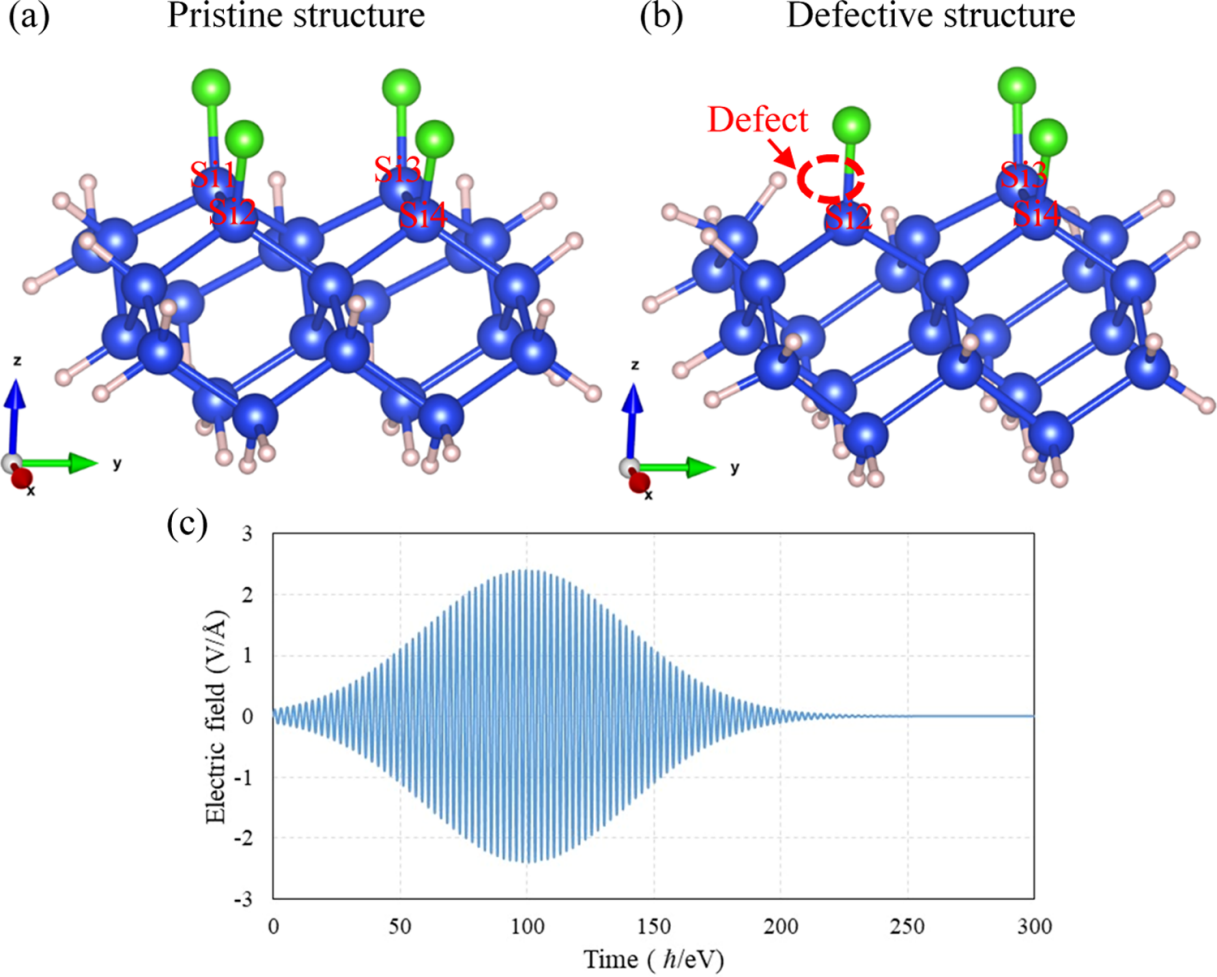
(a) Pristine structure and (b) defective structure of Cl–Si (100) surfaces under laser pulse irradiation for rt-TDDFT calculations, and (c) electric field of the laser with *E*_0_ = 2.4 V/Å and ω = 488 nm. Blue, green, and red atoms are Si, Cl, and H, respectively.

The effects of point defects on desorption dynamics were investigated by applying laser pulses using rt-TDDFT calculations. It should be noted that only four Si layers were used in the rt-TDDFT calculations since these calculations are significantly time-consuming. The size of cluster was examined in our previous work to mitigate its effects on calculation results.^32^Figure 4a,b depicts the pristine and defective structures used for calculations, respectively. The point defect was positioned at position 1, and particular attention was given to the desorption dynamics of nearby atoms at positions 2–4.

The applied laser pulse has a commercially used wavelength of 488 nm. The maximum electric field *E*_0_ of the laser ranged from 1.9 to 2.4 V/Å (corresponding to an intensity ranging from 4.79 × 10^13^ W/cm^2^ to 7.64 × 10^13^ W/cm^2^) in order to investigate the dynamics under different intensities. Figure 4c illustrates the time evolution of the electric field of the laser with *E*_0_ = 2.4 V/Å.

**Figure 5:**
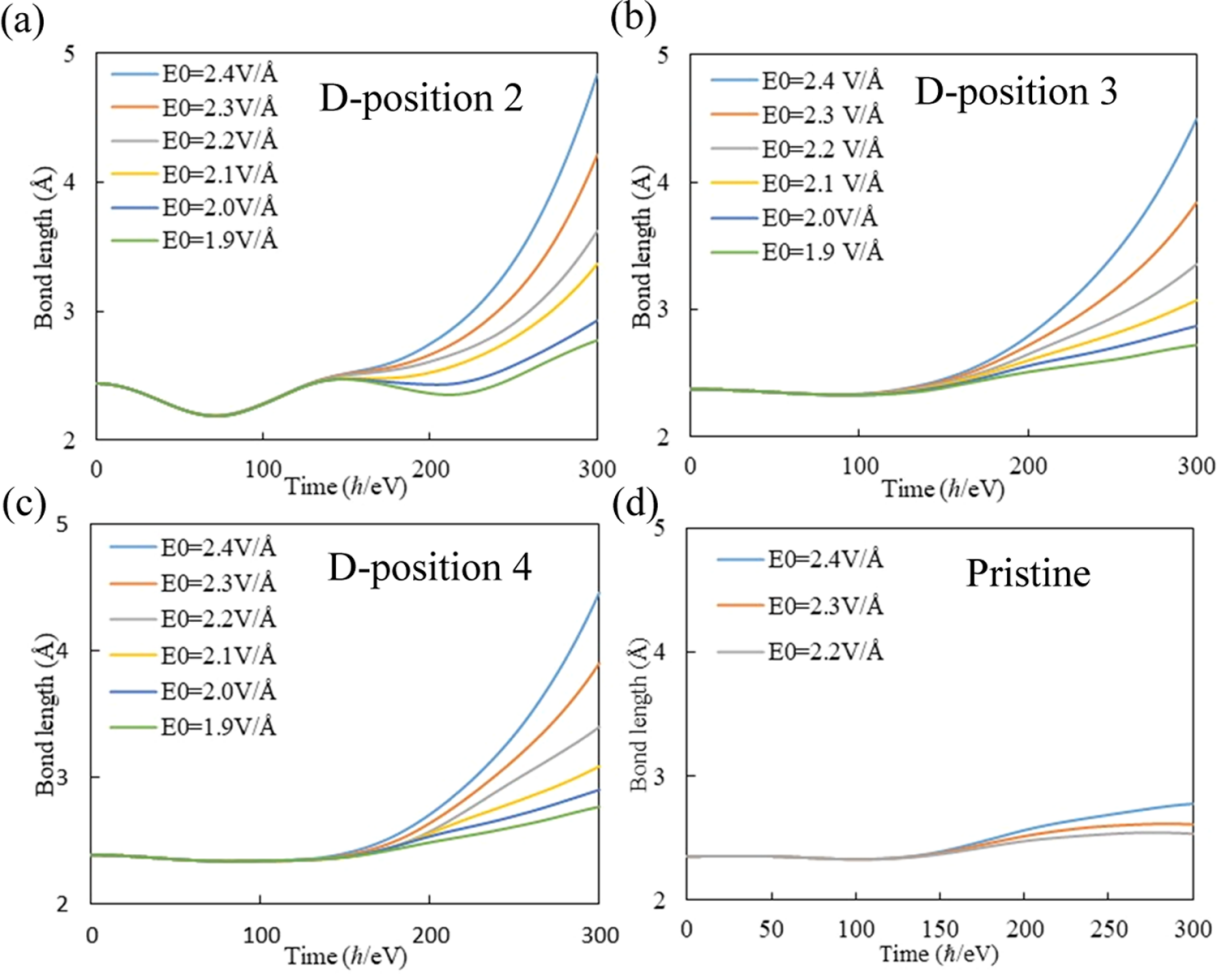
Time evolution of Si–Si bond lengths at (a) position 2, (b) position 3, and (c) position 4 of the defective structure and of (d) pristine structure. “D” represents the defective structure.

To illustrate the desorption process under laser pulses, the Si–Si bond lengths between Si atoms at positions 2–4 of the defective structure were plotted as shown in Figure 5a–c, respectively. Additionally, for comparative analysis, the bond length evolution of the pristine structure is included in Figure 5d. Note that for the pristine structure, the bond length evolution at position 2 sufficiently represents the desorption at other positions due to the structural symmetry. The initial Si–Si bond length between the first two Si layers is 2.35 Å, and it elongates under laser pulses. At higher laser intensities, the bond undergoes direct dissociation, resulting in the desorption of the SiCl molecule.

**Figure 6:**
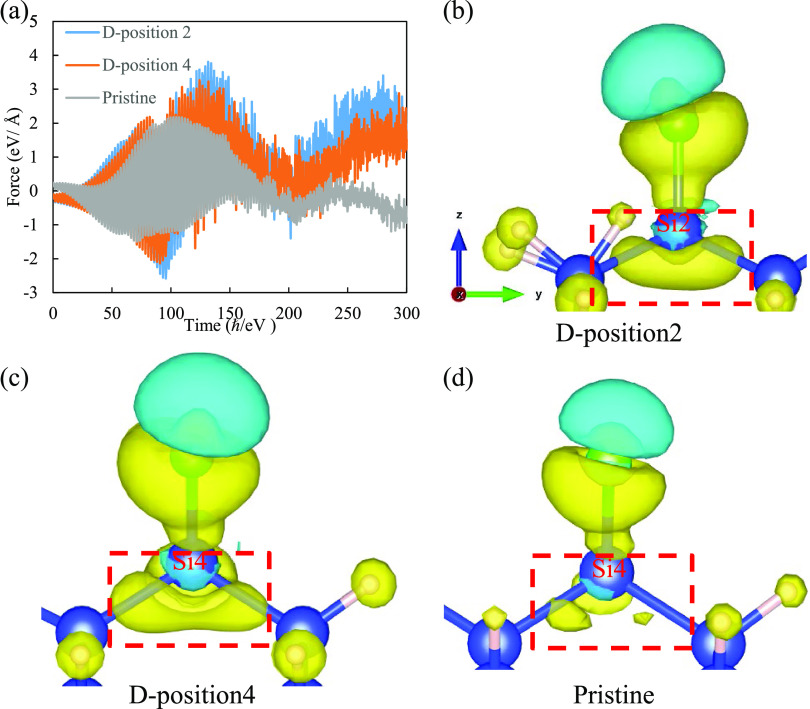
(a) *z*-Axis forces acting on SiCl at *E*_0_ = 2.4 V/Å, and electron density difference at *t* = 180 ℏ/eV (113 fs) at (b) position 2 and (c) position 4 of defective structure and (d) pristine structure. The yellow (cyan) isosurface represents the electron depletion (accumulation).

An important finding of this study is that the presence of point defects enhances the desorption of a nearby SiCl molecule. Specifically, at an electric field strength of *E*_0_ = 2.3 V/Å, the Si–Si bond lengths of the defective structure at positions 2–4 extend to 4.21, 3.90, and 3.84 Å, respectively, while those for the pristine structure reach only 2.61 Å. In the case of pristine structures, the Si–Si bond does not directly dissociate but instead oscillates along the bond direction when the electric field strength is below 2.6 V/Å.^32^ However, the introduction of a point defect lowers the intensity threshold for desorption from 2.6 to 2.1 V/Å. A similar phenomenon was also observed, where the desorption rate of atoms near defects on Si(111) surfaces is 2 orders of magnitude higher than that on pristine surfaces under laser irradiation.^50^ It is worth noting that this finding is not quite consistent with the calculated potential energy curves shown in Figure 2, where the desorption energy barrier at positions 3 and 4 appears to be at the same level as that of the pristine structure. The reason is that the existence of defects under laser irradiation introduces extra energy levels within the band gap. Besides, defects can enhance the laser–matter interaction by creating a local electric field around the defects. The enhanced interaction can cause more electron loss within the nearby Si–Si bonds (which will be illustrated in Figure 6), thereby facilitating the desorption of SiCl at positions 3 and 4.

To investigate the underlying mechanism of defect-enhanced desorption, we conducted force calculations acting on the Si atom of desorbed SiCl, and the results are presented in Figure 6a. Initially, rapid oscillating forces are observed, which can be attributed to the oscillating electric field of the laser. Subsequently, for defective structures, high repulsive forces arise between SiCl and the bulk. Notably, the magnitude of these repulsive forces for the defective structure is significantly higher than that for the pristine structure. For instance, at *t* = 180 ℏ/eV (113 fs), the forces at positions 2 and 4 of the defective structure are 1.14 and 1.36 eV/Å, respectively, whereas for the pristine structure, it is only 0.43 eV/Å, which is inadequate to rupture the Si–Si bond. The high value of the forces at defective structures can be traced back to the loss of electrons subjected to laser irradiation. To get a better visualization, the electron density difference at *t* = 180 ℏ/eV (113 fs) under a laser pulse with *E*_0_ = 2.4 V/Å was depicted. It is evident that the presence of defects leads to greater electron loss within the nearby Si–Si bonds compared to that of the pristine structure. This increased electron loss results in larger repulsive forces, as described by the force equation within Ehrenfest dynamics. Consequently, the attraction between SiCl and the substrate is diminished because of the altered ion–electron interaction term ∫*V*_ext_(**r**, **R**_*i*_)*n*(**r**, *t*)d**r**, where *V*_ext_ (**r**, **R**_*i*_) is the ionic core potential, and *n* (**r**,t) is the electron density.

### 3.3 Defect-Mediated Etching Regime

**Figure 7:**
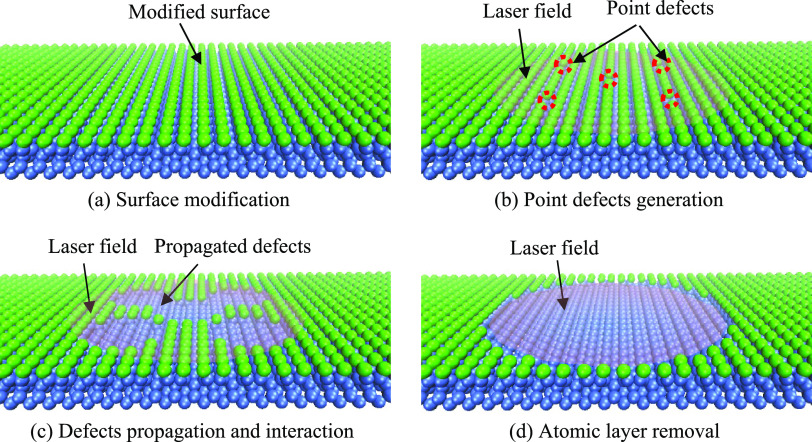
Schematic of the defect-mediated etching regime for the laser-induced ALE process.

The analysis presented above highlights the significant role of defects in the laser-stimulated desorption process, particularly in comparison to that of the pristine surface. Based on this analysis, a defect-mediated etching regime was proposed for the layer-by-layer removal process in laser-induced ALE of Si, as illustrated in Figure 7.

In the initial stage, laser pulse irradiation generates point defects on the topmost layer of Cl–Si(100). These point defects then propagate across the surface due to the defect-enhanced desorption mechanism. Eventually, the propagated defects interact with each other, leading to the complete removal of the entire topmost layer. It is important to note that the underlying second layer remains unaffected as long as the laser intensity remains below the desorption threshold of bulk Si, attributing to the fact that the underlying Si–Si bonds of the first layer are weakened by the presence of adsorbed Cl, while those of the second layer are less affected. The phenomenon of the unremoved second layer can be observed in the thinning of layered materials, like MoS_2_ and MoTe_2_, under laser irradiation.^18,19^

The defect-mediated etching regime bears similarity to the step-flow regime observed in the wet etching of Si(111) surfaces with NH_4_F. In wet etching, the material is predominantly removed along the atomic steps, which exhibit significantly higher etching rates compared to the pristine surface.^51,52^ This etching regime provides an explanation for the scanning tunneling microscopy (STM) images obtained during laser-induced ALE of GaAs(110), where defects grow along their edges.^36^ Moreover, there are also some relevant experimental studies in the existing literature that highlight the important role of defects in etching processes. For instance, previous research has shown that defects can substantially reduce the ablation threshold of materials, as exemplified by a 4-fold decrease in the ablation threshold of LiF when exposed to extreme ultraviolet laser irradiation.^53^ Furthermore, investigations have revealed that the photoetching rate in the vicinity of defects on the Si(111) surface surpasses that of defect-free regions by 2 orders of magnitude.^50^

Regarding potential methods for introducing point defects, the most accurate approach is the tip-based method, which includes techniques such as scanning tunneling microscopy (STM)-based and atomic force microscopy (AFM)-based methods. Successful applications of these methods can be found in the literature.^34,54^ However, a notable challenge associated with these tip-based methods is their relatively low manufacturing efficiency. Alternatively, other methods involve irradiation, such as electron and ion irradiations,^55−58^ which offer higher efficiency.

## 4 Conclusions

The role of point defects in the laser-induced ALE of Si was investigated by DFT and rt-TDDFT calculations at the atomic level. The presence of point defects was observed to enhance the desorption of nearby SiCl molecules from Si(100) surfaces. Specifically, it was found that the desorption energy barrier of the SiCl located at the dimer side of the point defect was reduced from 5.48 to 3.24 eV compared to the pristine surface. Additionally, the introduction of extra levels into the band gap was observed from the DOS diagram, and the intensity threshold required for the desorption of SiCl is lowered from 2.6 to 2.1 V/Å in the presence of point defects when using a laser pulse with a duration of 40 ℏ/eV and a wavelength of 488 nm. Based on this study, a defect-mediated etching regime was proposed to describe the layer-by-layer etching process. In this regime, point defects are initially created through a laser pulse irradiation. These defects then propagate and interact with each other, resulting in the removal of entire atomic layers. These findings provide fundamental insights for understanding the role of point defects in the laser-induced ALE process, which can contribute to the improvement of ALE techniques in the upcoming era of ACSM.

## References

[ref1] Kanarik K. J.; Lill T.; Hudson E. A.; Sriraman S.; Tan S.; Marks J.; Vahedi V.; Gottscho R. A. Overview of atomic layer etching in the semiconductor industry. J. Vac. Sci. Technol. A 2015, 33, 020802 10.1116/1.4913379.

[ref2] Carver C. T.; Plombon J. J.; Romero P. E.; Suri S.; Tronic T. A.; Turkot R. B. Atomic layer etching: An industry perspective. ECS J. Solid State Sci. Technol. 2015, 4, N5005 10.1149/2.0021506jss.

[ref3] Kanarik K. J.; Tan S.; Gottscho R. A. Atomic layer etching: rethinking the art of etch. J. Phys. Chem. Lett. 2018, 9, 4814–4821. 10.1021/acs.jpclett.8b00997. 30095919

[ref4] Oehrlein G. S.; Metzler D.; Li C. Atomic layer etching at the tipping point: an overview. ECS J. Solid State Sci. Technol. 2015, 4, N5041 10.1149/2.0061506jss.

[ref5] Zhang J.; Li Y.; Cao K.; Chen R. Advances in atomic layer deposition. Nanomanuf. Metrol. 2021, 5, 191–208. 10.1007/s41871-022-00136-8.

[ref6] Wu B.; Zhang Y.; Yi R.; Deng H. Tuning the plasma etching mode for the atomic-scale smoothing of single-crystal silicon. J. Phys. Chem. Lett. 2022, 13, 8580–8585. 10.1021/acs.jpclett.2c02121. 36073771

[ref7] Zhang J. F.; Ducree J. Proposition of atomic and close-to-atomic scale manufacturing Adv. Manuf. 2023 10.1007/s40436-023-00442-4.

[ref8] Hafeez H.; Xie W.; Luo X. In Atomic and Close-to-Atomic Scale Manufacturing: Status and Challenges, 28th International Conference on Automation and Computing (ICAC2023), 2023.

[ref9] Fang F. Z. The three paradigms of manufacturing advancement. J. Manuf. Syst. 2022, 63, 504–505. 10.1016/j.jmsy.2022.05.007.

[ref10] Fang F. Z. Atomic and close-to-atomic scale manufacturing: perspectives and measures. Int. J. Extreme Manuf. 2020, 2, 030201 10.1088/2631-7990/aba495.

[ref11] Fischer A.; Routzahn A.; George S. M.; Lill T. Thermal atomic layer etching: A review. J. Vac. Sci. Technol. A 2021, 39, 030801 10.1116/6.0000894.

[ref12] Tinacba E. J. C.; Isobe M.; Hamaguchi S. Surface damage formation during atomic layer etching of silicon with chlorine adsorption. J. Vac. Sci. Technol. A 2021, 39, 042603 10.1116/6.0001117.

[ref13] Ageev V. G.; Konov V. I.; Krechetov A. I.; Kuzmichov A. V.; Prokhorov A. M. In Excimer Laser Assisted Etching of Silicon Surface in Electronegative Gases, 1st International School on Laser Surface Microprocessing; SPIE, 1990; pp 5–17.

[ref14] Konov V. I. Laser in micro and nanoprocessing of diamond materials. Laser Photonics Rev. 2012, 6 (6), 739–766. 10.1002/lpor.201100030.

[ref15] Ishii M.; Meguro T.; Gamo K.; Sugano T.; Aoyagi Y. Digital etching using KrF excimer laser: approach to atomic-order-controlled etching by photo induced reaction. Jpn. J. Appl. Phys. 1993, 32 (12S), 6178 10.1143/JJAP.32.6178.

[ref16] Meguro T.; Ishii M.; Sugano T.; Gamo K.; Aoyagi Y. Control of the etching reaction of digital etching using tunable UV laser irradiation. Appl. Surf. Sci. 1994, 82, 193–199. 10.1016/0169-4332(94)90216-X.

[ref17] Eliceiri M.; Rho Y.; Li R.; Grigoropoulos C. P. Pulsed laser induced atomic layer etching of silicon. J. Vac. Sci. Technol. A 2023, 41, 022602 10.1116/6.0002399.

[ref18] Nagareddy V. K.; Octon T. J.; Townsend N. J.; Russo S.; Craciun M. F.; Wright C. D. Humidity-controlled ultralow power layer-by-layer thinning, nanopatterning and bandgap engineering of MoTe_2_. Adv. Funct. Mater. 2018, 28, 1804434 10.1002/adfm.201804434.

[ref19] Tessarek C.; Gridenco O.; Wiesing M.; Müssener J.; Figge S.; Sebald K.; Gutowski J.; Eickhoff M. Controlled laser-thinning of MoS_2_ nanolayers and transformation to amorphous MoOx for 2D monolayer fabrication. ACS Appl. Nano Mater. 2020, 3, 7490–7498. 10.1021/acsanm.0c01104.

[ref20] Rho Y.; Pei J.; Wang L.; Su Z.; Eliceiri M.; Grigoropoulos C. P. Site-selective atomic layer precision thinning of MoS_2_ via laser-assisted anisotropic chemical etching. ACS Appl. Mater. Interfaces 2019, 11, 39385–39393. 10.1021/acsami.9b14306. 31553575

[ref21] Nipane A.; Choi M. S.; Sebastian P. J.; Yao K.; Borah A.; Deshmukh P.; Jung Y.; Kim B.; Rajendran A.; Kwock K. W.; Zangiabadi A.; et al. Damage-free atomic layer etch of WSe_2_: A platform for fabricating clean two-dimensional devices. ACS Appl. Mater. Interfaces 2021, 13, 1930–1942. 10.1021/acsami.0c18390. 33351577

[ref22] Wang P.; Castelli M.; Fang F. F. Mechanism of photo-assisted atomic layer etching of chlorinated Si (111) surfaces: Insights from DFT/TDDFT calculations. Mater. Sci. Semicon. Process. 2023, 153, 107169 10.1016/j.mssp.2022.107169.

[ref23] Farigliano L. M.; Paredes-Olivera P. A.; Patrito E. M. Ab-initio molecular dynamics simulations of the reactivity of MoS_2_ towards F_2_ molecules: Implications for etching processes. Appl. Surf. Sci. 2023, 607, 154637 10.1016/j.apsusc.2022.154637.

[ref24] Farigliano L. M.; Paredes-Olivera P. A.; Patrito E. M. Initial steps of oxidative etching of MoS_2_ basal plane induced by O_2_. J. Phys. Chem. C 2020, 124, 13177–13186. 10.1021/acs.jpcc.0c02141.

[ref25] Huard C. M.; Zhang Y.; Sriraman S.; Paterson A.; Kanarik K. J.; Kushner M. J. Atomic layer etching of 3D structures in silicon: Self-limiting and nonideal reactions. J. Vac. Sci. Technol. A 2017, 35, 031306 10.1116/1.4979661.

[ref26] Vella J. R.; Humbird D.; Graves D. B. Molecular dynamics study of silicon atomic layer etching by chlorine gas and argon ions. J. Vac. Sci. Technol. B 2022, 40, 023205 10.1116/6.0001681.

[ref27] Zhang S.; Huang Y.; Tetiker G.; Sriraman S.; Paterson A.; Faller R. Computational modelling of atomic layer etching of chlorinated germanium surfaces by argon. Phys. Chem. Chem. Phys. 2019, 21, 5898–5902. 10.1039/C9CP00125E. 30809623

[ref28] Kanarik K. J.; Tan S.; Yang W.; Kim T.; Lill T.; Kabansky A.; Hudson E. A.; Ohba T.; Nojiri K.; Yu J.; Wise R.; et al. Predicting synergy in atomic layer etching. J. Vac. Sci. Technol. A 2017, 35, 05C302 10.1116/1.4979019.

[ref29] George S. M. Mechanisms of thermal atomic layer etching. Acc. Chem. Res. 2020, 53, 1151–1160. 10.1021/acs.accounts.0c00084. 32476413

[ref30] Fang C.; Cao Y.; Wu D.; Li A. Thermal atomic layer etching: Mechanism, materials and prospects. Prog. Nat. Sci. 2018, 28, 667–675. 10.1016/j.pnsc.2018.11.003.

[ref31] Wang P.; Fang F. F. Real-time time-dependent DFT study of laser-enhanced atomic layer etching of silicon for damage-free nanostructure fabrication. J. Appl. Phys. 2022, 132, 144303 10.1063/5.0109818.

[ref32] Wang P.; Fang F. F. Ab initio simulations of ultrashort laser pulse interaction with Cl–Si (100): implications for atomic layer etching. Phys. Chem. Chem. Phys. 2023, 25, 20871–20879. 10.1039/D3CP02388E. 37522855

[ref33] Hinuma Y.; Toyao T.; Kamachi T.; Maeno Z.; Takakusagi S.; Furukawa S.; Takigawa I.; Shimizu K. I. Density functional theory calculations of oxygen vacancy formation and subsequent molecular adsorption on oxide surfaces. J. Phys. Chem. C 2018, 122, 29435–29444. 10.1021/acs.jpcc.8b11279.

[ref34] Dwyer K. J.; Dreyer M.; Butera R. E. STM-induced desorption and lithographic patterning of Cl-Si (100)-(2 × 1). J. Phys. Chem. A 2019, 123, 10793–10803. 10.1021/acs.jpca.9b07127. 31725292

[ref35] Lyons J. L.; Wickramaratne D.; Van de Walle C. G. A first-principles understanding of point defects and impurities in GaN. J. Appl. Phys. 2021, 129 (11), 111101 10.1063/5.0041506.

[ref36] Han B. Y.; Weaver J. H. Laser interaction with Br-GaAs (110): Etching and atomic desorption. Phys. Rev. B 1998, 58, 10981 10.1103/PhysRevB.58.10981.

[ref37] Feng L. P.; Su J.; Liu Z. T. Effect of vacancies on structural, electronic and optical properties of monolayer MoS_2_: a first-principles study. J. Alloys Compd. 2014, 613, 122–127. 10.1016/j.jallcom.2014.06.018.

[ref38] Wang J.; Fang F.; An H.; Wu S.; Qi H.; Cai Y.; Guo G. Laser machining fundamentals: micro, nano, atomic and close-to-atomic scales. Int. J. Extreme Manuf. 2023, 5, 012005 10.1088/2631-7990/acb134.

[ref39] Giannozzi P.; Baroni S.; Bonini N.; Calandra M.; Car R.; Cavazzoni C.; Ceresoli D.; Chiarotti G. L.; Cococcioni M.; Dabo I.; Dal Corso A.; et al. QUANTUM ESPRESSO: a modular and open-source software project for quantum simulations of materials. J. Phys.: Condens. Matter 2009, 21, 395502 10.1088/0953-8984/21/39/395502. 21832390

[ref40] Perdew J. P.; Burke K.; Ernzerhof M. Generalized gradient approximation made simple. Phys. Rev. Lett. 1996, 77, 3865 10.1103/PhysRevLett.77.3865. 10062328

[ref41] Monkhorst H. J.; Pack J. D. Special points for Brillouin-zone integrations. Phys. Rev. B 1976, 13, 5188 10.1103/PhysRevB.13.5188.

[ref42] Andrade X.; Strubbe D.; De Giovannini U.; Larsen A. H.; Oliveira M. J.; Alberdi-Rodriguez J.; Varas A.; Theophilou I.; Helbig N.; Verstraete M. J.; Stella L.; et al. Real-space grids and the Octopus code as tools for the development of new simulation approaches for electronic systems. Phys. Chem. Chem. Phys. 2015, 17, 31371–31396. 10.1039/C5CP00351B. 25721500

[ref43] Goedecker S.; Teter M.; Hutter J. Separable dual-space Gaussian pseudopotentials. Phys. Rev. B 1996, 54 (54), 1703 10.1103/PhysRevB.54.1703. 9986014

[ref44] Castro A.; Marques M. A.; Rubio A. Propagators for the time-dependent Kohn–Sham equations. J. Chem. Phys. 2004, 121, 3425–3433. 10.1063/1.1774980. 15303905

[ref45] Casagrande D.; Srivastava G. P.; Ferraz A. C. Theoretical calculations for Si (001)-(2× 1) Cl. Surf. Sci. 1998, 402, 653–657. 10.1016/S0039-6028(97)00929-1.

[ref46] Pavlova T. V.; Shevlyuga V. M.; Andryushechkin B. V.; Eltsov K. N. Chlorine insertion and manipulation on the Si (100)-2× 1-Cl surface in the regime of local supersaturation. Phys. Rev. B 2020, 101, 235410 10.1103/PhysRevB.101.235410.

[ref47] Matsunaga K.; Tanaka T.; Yamamoto T.; Ikuhara Y. First-principles calculations of intrinsic defects in Al_2_O_3_. Phys. Rev. B 2003, 68, 085110 10.1103/PhysRevB.68.085110.

[ref48] Hashibon A.; Elsässer C. First-principles density functional theory study of native point defects in Bi_2_Te_3_. Phys. Rev. B 2021, 84, 144117 10.1103/PhysRevB.84.144117.

[ref49] Majidi S.; Achour A.; Rai D. P.; Nayebi P.; Solaymani S.; Nezafat N. B.; Elahi S. M. Effect of point defects on the electronic density states of SnC nanosheets: first-principles calculations. Results Phys. 2017, 7, 3209–3215. 10.1016/j.rinp.2017.08.049.

[ref50] Chen X. H.; Polanyi J. C.; Rogers D. Photoetching of Si (111)-(7 × 7) studied by STM. Surf. Sci. 1997, 376, 77–86. 10.1016/S0039-6028(96)01403-3.

[ref51] Hines M. A. The picture tells the story: using surface morphology to probe chemical etching reactions. Int. Rev. Phys. Chem. 2001, 20, 645–672. 10.1080/01442350110071966.

[ref52] Zhou H.; Fu J.; Silver R. M. Time-resolved kinetic Monte-Carlo simulation study on Si (111) etching. J. Phys. Chem. C 2007, 111, 3566–3574. 10.1021/jp060941j.

[ref53] Cherednikov Y.; Inogamov N. A.; Urbassek H. M. Influence of defects on extreme ultraviolet laser ablation of LiF. Phys. Rev. B 2013, 88, 134109 10.1103/PhysRevB.88.134109.

[ref54] Custance O.; Perez R.; Morita S. Atomic force microscopy as a tool for atom manipulation. Nat. Nanotechnol. 2009, 4, 803–810. 10.1038/nnano.2009.347. 19966795

[ref55] Krasheninnikov A. V.; Nordlund K.; Sirviö M.; Salonen E.; Keinonen J. Formation of ion-irradiation-induced atomic-scale defects on walls of carbon nanotubes. Phys. Rev. B 2001, 63, 245405 10.1103/PhysRevB.63.245405.

[ref56] Kotakoski J.; Krasheninnikov A. V.; Kaiser U.; Meyer J. C. From point defects in graphene to two-dimensional amorphous carbon. Phys. Rev. Lett. 2011, 106, 105505 10.1103/PhysRevLett.106.105505. 21469806

[ref57] Tuomisto F.; Saarinen K.; Look D. C.; Farlow G. C. Introduction and recovery of point defects in electron-irradiated ZnO. Phys. Rev. B 2005, 72, 085206 10.1103/PhysRevB.72.085206. 9980192

[ref58] Crocombette J. P.; Proville L. Thermal conductivity degradation induced by point defects in irradiated silicon carbide. Appl. Phys. Lett. 2011, 98, 191905 10.1063/1.3589358.

